# Profiling conserved transcription factor binding motifs in *Phaseolus vulgaris* through comparative genomics

**DOI:** 10.1186/s12864-025-11309-2

**Published:** 2025-02-20

**Authors:** Liudmyla Kondratova, C. Eduardo Vallejos, Ana Conesa

**Affiliations:** 1 Genetics & Genomics Graduate Program, University of Florida, Gainesville, FL USA; 2 Horticultural Sciences Department, University of Florida, Gainesville, FL USA; 3 Institute for Integrative Systems Biology, Spanish National Research Council, Paterna, Spain

**Keywords:** Transcription factor binding sites conservation, Regulatory networks, Common bean, *P. vulgaris*, Comparative genomics, Non-model organisms

## Abstract

Common bean (*Phaseolus vulgaris*), a staple food in Latin America and Africa, serves as a vital source of energy, protein, and essential minerals for millions of people. However, genomics knowledge that breeders could leverage for improvement of this crop is scarce. We have developed and validated a comparative genomics approach to predict conserved transcription factor binding sites (TFBS) in common bean and studied gene regulatory networks. We analyzed promoter regions and identified TFBS for 12,631 bean genes with an average of 6 conserved motifs per gene. Moreover, we discovered a statistically significant relationship between the number of conserved motifs and amount of available experimental evidence of gene regulation. Notably, ERF, MYB, and bHLH transcription factor families dominated conserved motifs, with implications for starch biosynthesis regulation. Furthermore, we provide gene regulatory data as a resource that can be interrogated for the regulatory landscape of any set of genes. Our results underscore the significance of TFBS conservation in legumes and aligns with the notion that core genes often exhibit a more conserved regulatory makeup. The study demonstrates the effectiveness of a comparative genomics approach for addressing genome information gaps in non-model organisms and provides valuable insights into the regulatory networks governing starch biosynthesis genes that can support crop improvement programs.

**Supplementary Information:**

The online version contains supplementary material available at 10.1186/s12864-025-11309-2.

## Background

Global population pressure combined with climate change threatens food security for the human population [1]. Genetic improvement of crops is a recognized strategy to address the challenges posed by these adverse global factors [2, 3] and to develop crops with higher yields, resistance to pests, and the ability to adapt to changing climatic conditions. Although a substantial number of crop species count with a draft of their genome (https://phytozome-next.jgi.doe.gov/), most of them do not have the high level of detailed genome annotation as model organisms. Many crops possess vast pools of genetic diversity and valuable traits for adaptation to adverse environments. However, limited genome annotation hinders the application of genome knowledge-based solutions for crop improvement, which could help us overcome food security challenges imposed by climate change.

Genetic improvement harnesses natural or induced genetic variation, which is due to differences in gene product functionality, gene expression patterns, or both. Transcription factors (TF) are proteins that regulate the spatiotemporal patterns of gene expression through their ability to bind specific DNA sequences known as *cis*-regulatory elements [4]. Regulatory networks composed of transcription factors and their target genes govern various plant processes, including growth, development, stress responses, and metabolic functions - traits critical for improving yield and adaptability [5–7]. Several experimental methods have been developed to localize and characterize the *cis*-regulatory elements or transcription factor binding sites (TFBS) [8]. Experimental identification of TFBS in both plant and animal model systems has led to the creation of extensive databases for TFs and TFBS [9–11]. These databases, in turn, have fostered the development of computational prediction methods to expand the TFBS space into non-model organisms [12–14]. However, in the absence of additional information, computational methods for inferring TFBS in non-model species suffer from high false positive rates, a problem that challenges their utility for studying gene regulatory networks in these species.

The possibility of computational prediction of TFBS is supported by the observed conservation of regulatory elements in both plants and vertebrates [15, 16]. Several studies have demonstrated the functional importance of highly conserved TFBS across evolutionarily closely related plant species [17]. Kumari & Ware (2013) analyzed the homologies of binding motifs and DNA free energy profiles to develop a prediction model for conserved core promoter elements across monocots and dicots. Moreover, ChIP-seq-based comparisons of binding sites for MADS-box transcription factors in *Arabidopsis thaliana* (FLC) and *Arabis alpina* (PEP1), which belong to different lineages of the Brassicaceae family, revealed that approximately 14% of PEP1 binding sites are conserved between the two species [18]. Genes with conserved binding sites exhibited more significant changes in expression in *flc/pep1* mutants, indicating the regulatory potential of conserved binding sites. The study concluded that conservation assessment is a powerful approach for identifying core genes regulated by a transcription factor. Similarly, other studies have employed conservation-based strategies to identify *cis*-acting elements in *Drosophila* [19] and *Zea mays* [20].

In this work, we deploy a novel comparative genomics approach designed to identify conserved TFBS in the common bean, *Phaseolus vulgaris*. The common bean is a staple food for millions of people in Latin America and Africa where it serves as an indispensable source of energy (starch), protein, fiber, and essential minerals [21]. Due to their nutritional value and adaptability, beans are considered a potential solution to the current food and climate crisis [22]. However, TFBS annotation in the *P. vulgaris* genome is lacking as is the description or characterization of gene regulatory networks. We employed computational methods to predict TFBS in the common bean and utilized comparative genomics of promoter regions of orthologous genes in *Vigna angularis*, *V. radiata*, and *Glycine max* to identify conserved TFBSs in *P. vulgaris*. We discerned essential information required to characterize the regulatory programs that govern gene expression in the common bean through the analysis of the quantity, distribution, and frequencies of these TFBSs. Specifically, we identified a core TF network involved in starch biosynthesis and their genetic variation across different genetic pools and accessions. Our pipeline represents a valuable approach for hypothesis generation in the study of the regulatory landscape of gene expression in the common bean and can serve to inform future breeding programs aimed at enhancing crop resilience to changing environmental conditions. This approach can easily be implemented for many other crop species with available genome sequence.

## Methods

### Extraction of *P. vulgaris* promoter regions

While the most recent version of the common bean reference genome is v2.1, at the time of this project Ensembl Plants (https://plants.ensembl.org) was using v1.0 for orthology definition [23]. Therefore, we selected promoter regions with high similarity between the two references. Specifically, sequence regions (-2000 to + 200 relative to the TSS) were extracted from both references and aligned using Minimap2. The selection of this promoter region size for the analysis was based on the fact that some of the previous research of conserved TFBSs was done using the same span [16]. While we acknowledge that restricting analysis to these defined regions overlooks distal and intragenic sites, expanding the promoter spans could result in higher false discovery rates.

Promoters of genes with 90% or more identity between the references were selected for further analysis. Selected promoters were used to identify conserved TFBSs. Further, predicted conserved TFBSs were located within the promoter regions in v2.1.

### Functional annotation of *P. vulgaris* genes

The protein-coding sequences of *P. vulgaris* were downloaded from Ensembl Plants and functionally annotated using the Blast2GO software [24] available through OmixBox. Blast2GO uses blast hits to retrieve Gene Ontology (GO) terms for the provided sequences. The default parameters were used on the plant database to obtain the annotations.

### Identifying similarity in promoter regions of orthologous genes

#### Obtaining orthologous genes from Ensembl plants

The orthologous genes for *V. radiata* [25], *V. angularis* [26], and *G. max* [27] were obtained from Ensembl Plants (release 56) for comparative analysis with *P. vulgaris*. Ensembl Plants is a sub-portal of Ensembl Genomes– a comprehensive and collaborative platform that provides access to genome data and functional information for a wide range of plant species [28]. Ensembl Genomes is a gene-focused phylogenetic resource that employs Ensembl Compara GeneTrees to call orthologous genes [29]. The orthologs are derived from pre-computed protein gene trees. These gene trees are structured using the TreeFam HMM (Hidden Markov Models) library, which is based on the Panther database. Following the extraction of orthologs, multiple alignments were generated for each gene family, leading to the construction of phylogenetic trees.

The identification of orthologs and their respective orthology type hinges on gene pairwise relations within each gene tree. Ensembl employs two independent metrics for assessing the quality of orthology: the gene order conservation (GOC) score and the whole genome alignment (WGA) score. The GOC score leverages the likelihood that orthologous genes are syntenic, assessing the number of four closest neighbors of the target gene that match across species. Meanwhile, the whole genome alignment score employs pairwise whole genome alignments to ascertain coverage over orthologue pairs. This metric incorporates a weighted sequence similarity evaluation spanning the exons and introns of potential orthologs. The selection of high confidence orthologs culminates in those exceeding a 25% identity and meeting both GOC and WGA thresholds. One-to-one and one-to-many orthologs were selected for the analysis.

#### Identifying similarities in promoters of orthologous genes

To identify similarity in the promoter regions, sequences corresponding to 2000 nt upstream and 200 nt downstream of the TSS of all *P. vulgaris* genes were aligned to the promoters of the orthologous genes using the k-means aligner Minimap2 [30]. While multiple sequence aligners are commonly used for conservation analysis [31, 32], Minimap2 was found to identify about four times more similar regions between promoters than the multiple aligner MUSCLE (data not shown). We ran Minimap2 with options *-c* to generate the cigar and *secondary = yes* to capture multiple alignments. The rest of the Minimap2 parameters were left to default.

Alignments corresponding to orthologous pairs were selected for further analysis. In cases where a gene had homologous promoters in multiple orthologs (for one-to-many cases), the alignment of the ortholog with the highest protein homology was selected.

#### Analysis of promoter similarities

The coverage of alignments across promoter, i.e. the number of genes with sequence similarity at each promoter position, was calculated by first extracting into a bed file the alignment starting and ending positions of each promoter. The chromosome name was set to a species name and the three files were combined. Then, we used *bedtools coverage* [33] to calculate how often each of the positions regarding TSS is within the alignment span.

To calculate the relationship between the percent similarity of the protein sequence and the proportion of orthologs with similar promoter sequences, we initially grouped orthologous pairs based on the percent similarity of their protein sequences into 100 bins. Each bin represents a rounded integer percentage of homology, ranging from 0 to 100. Then, we calculated the proportion of genes with similar promoters within each group. Finally, we used a weighted linear regression to account for unequal gene numbers within each homology percentage group.

#### Conservation test

We retrieved 338 *P. vulgaris* TFBSs representing 40 families from the Plant Transcription Factor Database [34]. Furthermore, we used FIMO [13] to conduct motif enrichment analyses on the previously extracted promoter regions using these plant TF binding motifs and a 0-order Markov background model of promoter regions. To identify conserved motifs in promoters of *P. vulgaris* and each of the other three species, we first selected orthologs with similar promoter sequences, and then looked for the exact sequence match on the same strand and within 100 nucleotides of the original TFBS.

#### Functional enrichment analysis

Functional enrichment analysis of genes with conserved motifs between common bean and each of the three species was performed with GOATOOLS [35]. Genes with at least one conserved TFBS were selected for the analysis regardless of the promoter’s similarity level. For this, *P. vulgaris* genes were functionally annotated with Blast2GO (Conesa et al., 2005) using default parameters.

To identify biological functions that were overrepresented within genes having conserved motifs for a particular TF family, we conducted a binomial test to compare the frequency of a given biological role within this subset of genes to its frequency on a genome-wide level. To control for false discoveries, we applied FDR method for multiple-testing correction to *p* values. The adjusted *p.value* significance threshold was set to 0.05.

#### Accessible chromatin regions analysis

We utilized an open-source dataset obtained from *P. vulgaris* leaves to identify accessible chromatin regions (ACRs) [36]. To perform this task, we selected ACRs located within promoters selected for this study and then performed a chi-square test using the number of conserved nucleotides inside and outside of ACRs to test the hypothesis that nucleotide sequences located within ACRs were conserved more often than those outside of ACRs.

#### Genetic variants analysis

Raw whole genome sequence of 126 common bean individual plants (NCBI, PRJNA471678) were processed with Trimmomatic [37] and mapped to the v2.1 common bean reference genome [38] with BWA aligner [39]. Further, the alignments were subjected to gatk MarkDuplicates to tag duplicated reads [40]. The alignments of starch biosynthesis genes were extracted, and BCFtools mpileup and call functions were used to call variants. Bedtools intersect was used to identify variants located within the conserved TFBSs.

## Results

### Overview of our computational approach

**Fig. 1:**
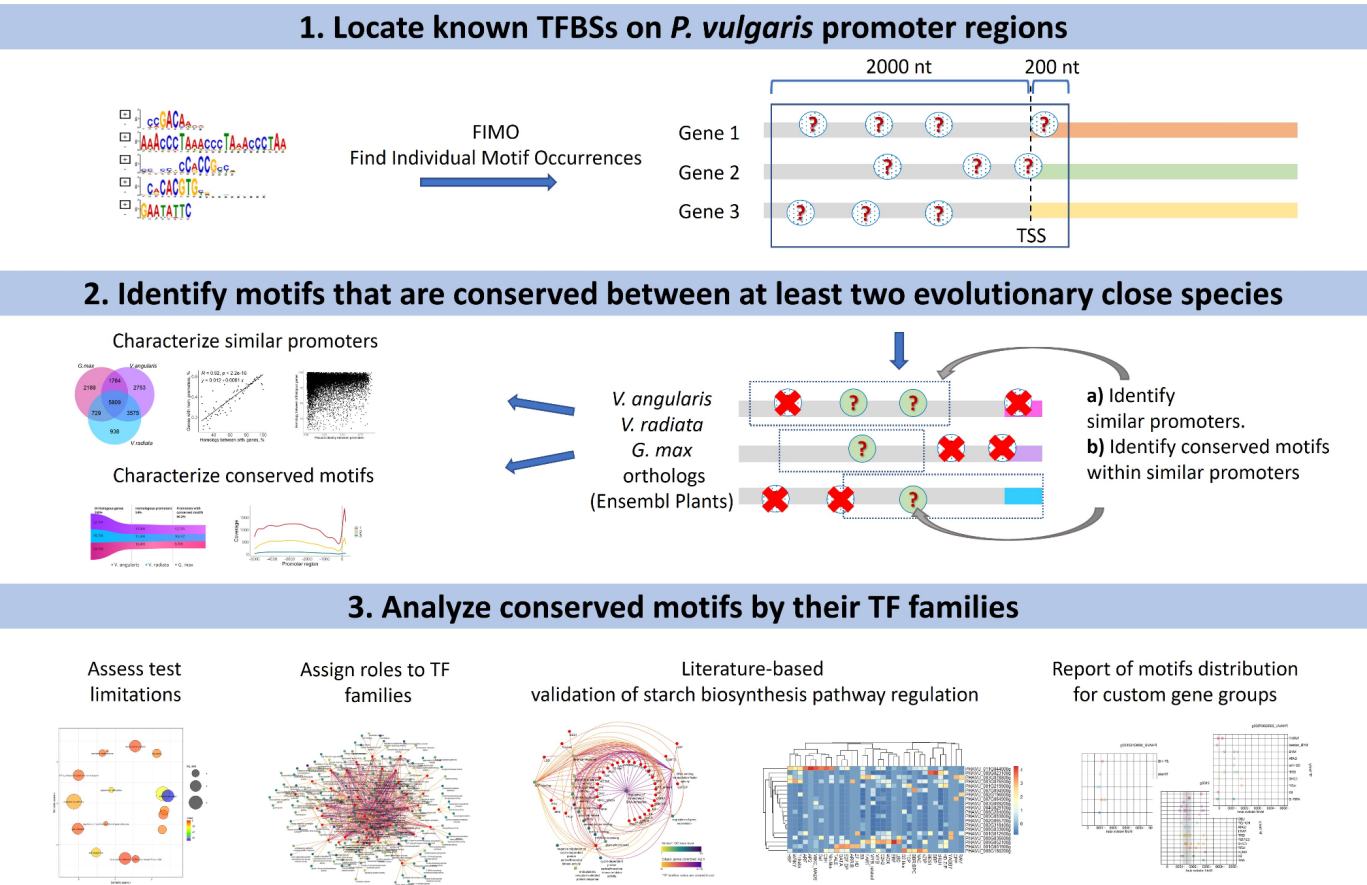
Experimental workflow

We utilized the concept of regulatory network conservation to identify evolutionarily constrained regions in gene promoters as potential TFBSs (Fig. 1). First, we identified known TFBSs specific to the common bean within the upstream sequences of TSS of annotated genes using FIMO [13]. Subsequently, we employed a sequence mapping approach to detect similar regions within the promoters of genes orthologous to common bean genes in *V. angularis*, *V. radiata*, and *G. max*. These regions were then examined for the presence of predicted binding motifs; this approach is based on previous observations that TFBS tend to be under purifying selection in closely related species [41]. The conserved motifs were carefully analyzed to identify potential biases in the approach, assign putative biological roles to transcription factor families, and validate our findings. Further, we identified genetic variation within wild and domesticated Andean and Mesoamerican accessions. Additionally, we provide an R script that enables users to obtain TFBS annotation data for a specific set of genes in the form of a report that includes comprehensive information on the presence, distribution, and co-occurrence of conserved binding sites and their putative transcription factors (Fig. 1).

Firstly, known transcription factor binding sites in the promoter regions of common bean genes were predicted using computational methods. Next, homologous regions between promoters of orthologous genes in *V. angularis*, *V. radiata*, and *G. max* were identified by mapping. The high-similarity promoter regions were then scanned for the presence of previously predicted binding motifs. Conserved motifs were analyzed to assess potential biases, assign roles to transcription factor families, and validate findings. Finally, data can be queried for specific genes and a graphical representation of the presence, distribution, and co-occurrence of conserved motifs and transcription factors at promoters is provided.

### Promoter regions of orthologous genes show increased sequence similarity

We aligned the promoter regions of bean genes with those of their ortholog genes in each of the three related species (*V. angularis*, *V. radiata*, *G. max*), designating as similar those promoters that displayed significant alignments (see Methods). To exclude the possibility of identifying similar promoters due to spurious alignments, we shuffled the orthology assignment between *P. vulgaris* and the three other species to create false orthologs and repeated the alignment. No spurious alignments were identified for *G. max* and *V. radiata* orthologs and only 2 similar promoters were identified for randomly assigned *V. angularis* orthologs. These results indicated high specificity in the similarity among promoters of orthologous genes.

**Fig. 2:**
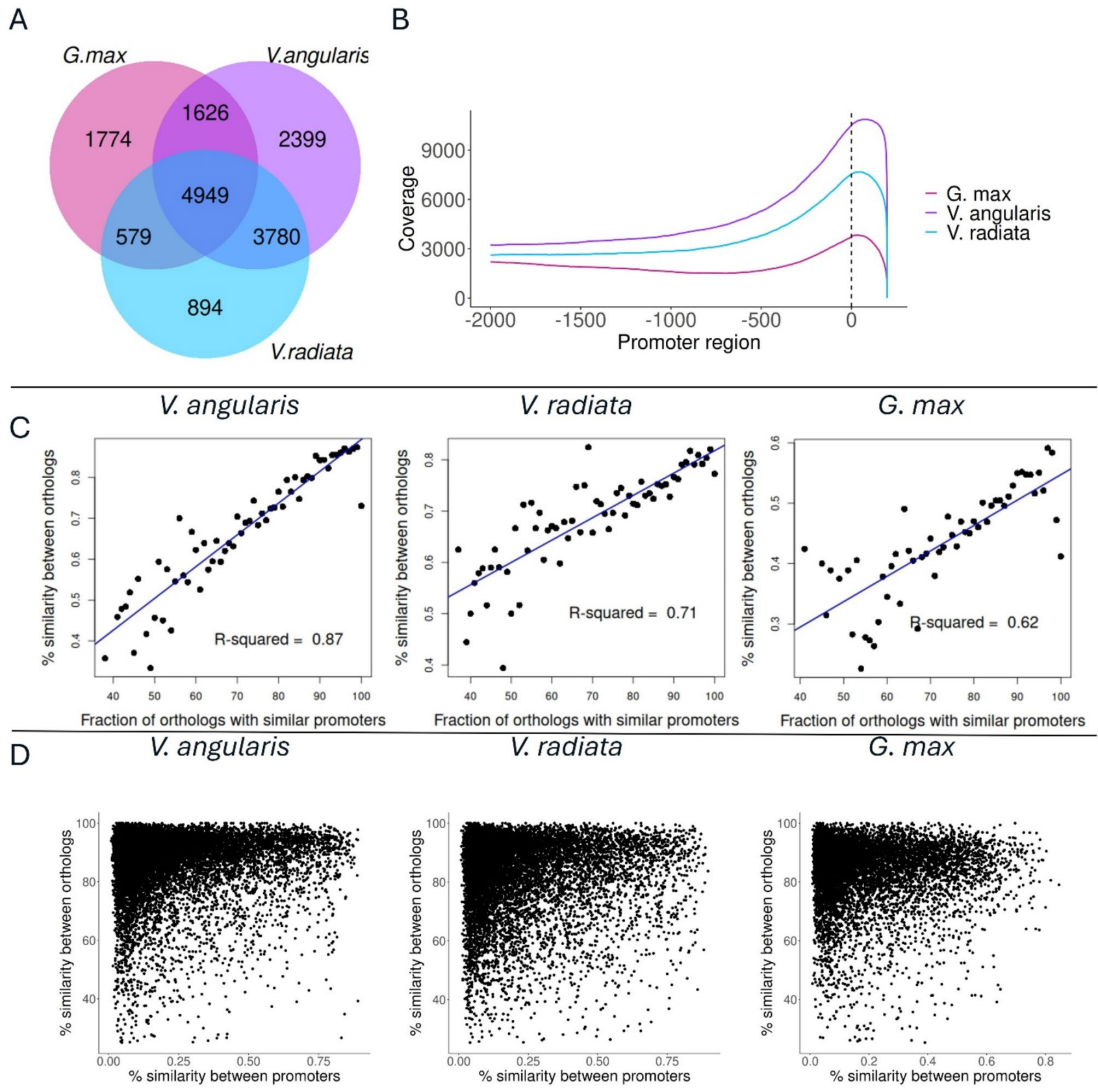
Key features of similar promoter regions. **(A)** Number of *P. vulgaris* genes that have similar promoter regions in other species. **(B)** Distribution of similarity levels in similar promoters along the length of promoter region. The Y axis indicates the number of gene promoters with successful mapping in *P.vulgaris*, while the X axis indicates the nucleotide position upstream from the *P. vulgaris* TSS. **(C)** Correlation between the percent homology level of orthologous genes and the proportion of genes that exhibit similar promoter regions. **(D)** Relationship between the percent similarity of orthologous genes and the percent identity of their promoter regions

Out of the 23,811 genes selected for the analysis, 12,754 genes had similarity in their promoter regions with the corresponding sequence of their orthologous genes in *V. angularis*, 10,202 genes in *V. radiata*, and only 8,928 with *G. max* orthologs; average percent identities were 0.22, 0.22, and 0.18, respectively (Fig. 2A). The alignment spanned an average of 878, 835, and 634 nucleotides across the query common bean promoters for *V. angularis*, *V. radiata*, and *G. max*, respectively.

Next, we investigated the distribution of sequence similarity levels in similar promoters of orthologous genes as a function of the distance to the TSS by computing the per-nucleotide accumulated similarity level for all similar promoters. We found that the highest level of similarity was located near the TSS with decreasing similarity as the distance to the TSS increased (Fig. 2B). In addition, the number of promoter regions with similar nucleotides around TSS decreased as the phylogenetic distance between species increased, with *V. angularis*, the closest species to common bean, showing the greatest similarity rates and *G. max*, furthest in the phylogenetic tree, the lowest (Fig. 2B). The distribution of coding regions of the upstream genes in reference to the position of the TSS of the downstream gene is featured in Supplementary Fig. 1.

Linear regression analysis showed that the similarity percentage between orthologous genes is a predictor of the presence of a similar promoter, with R-squared values ranging from 0.62 to 0.87 (Fig. 2C). Additionally, although we did not find a linear relationship between the percent similarity of protein sequences of orthologous genes and the percent identity of their promoter regions, we did observe that orthologous genes with high similarity are more likely to have similar promoters, suggesting that conserved genes are more likely to have conserved promoters (Fig. 2D).

From these analyses we concluded that promoter regions of highly conserved genes tend to display highly similar sequences around their TSS, motivating the further analysis of conserved regulatory signals.

### Conserved TFBSs are preferentially located near TSS

**Fig. 3:**
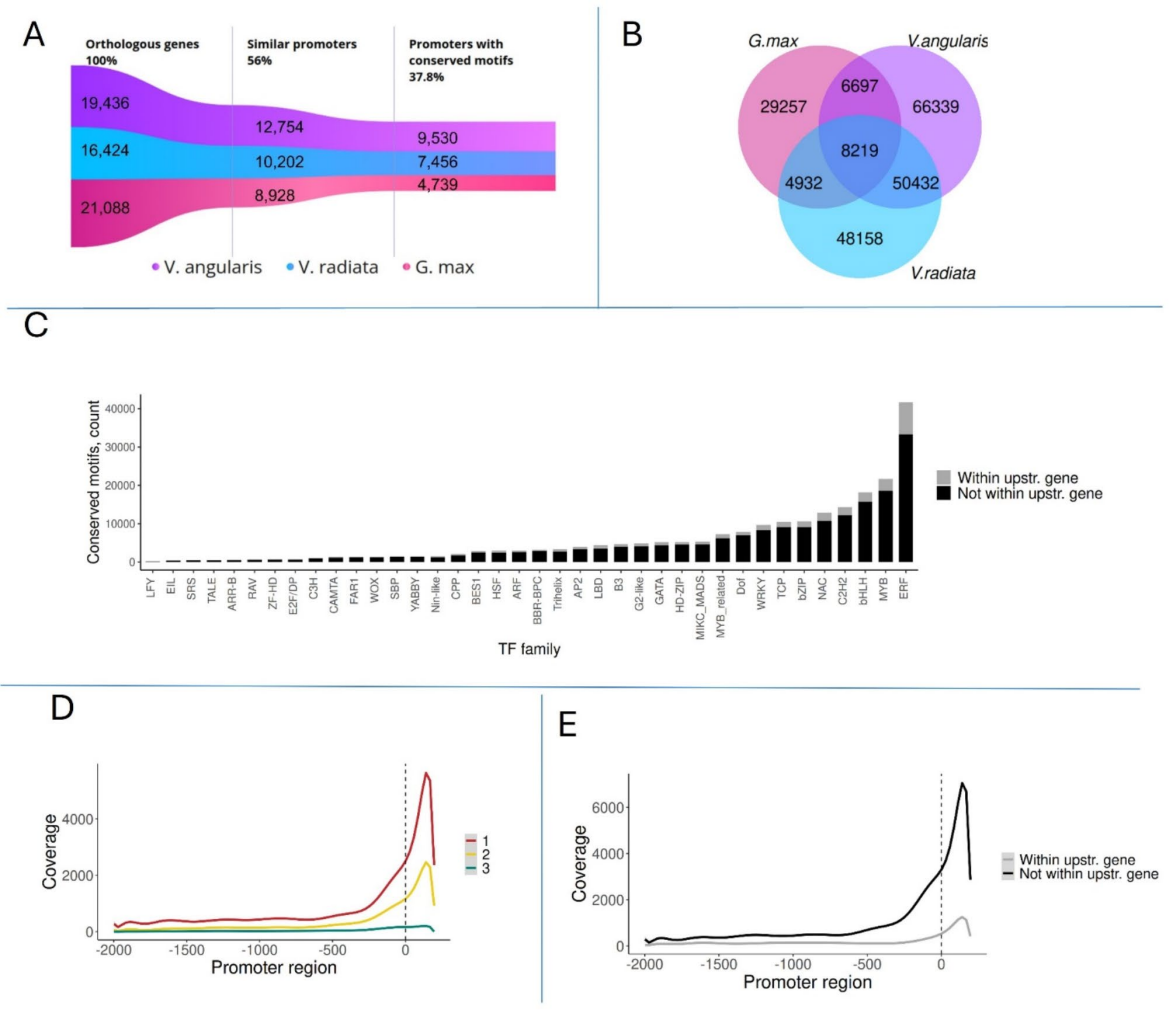
Characteristics of conserved TFBSs. **(A)** Number of *P. vulgaris* genes that have orthologs, similar promoters, and conserved TFBSs with each of the compared species. **(B)** Number of shared conserved TFBSs between all studied species. **C)** Number of conserved TFBSs by TF families. TFBSs present in *P.vulgaris* and at least one other species are included. Each bar represents the number of TFBS belonging to a specific family. The black portion of the bar indicates the number of conserved sites that do not overlap with annotated upstream gene, while the portion area shows the number of sites located within the body of an upstream gene. **(D)** Distribution in the number of conserved TFBSs across TSS upstream regions by their conservation level. A higher conservation level indicates that a TFBS is present in *P. vulgaris* promoter and on promoter regions of multiple studied species. For example, conservation level of 1 means that the TFBS is conserved between *P. vulgaris* and one other species, while conservation level of 3 indicates that the site is present in all studied species. **(E)** Distribution of conserved TFBSs across promoters is assessed based on their overlap with annotated features. The gray line represents the number of conserved sites located within the body of the gene, excluding the flanking genes

Using FIMO, we identified 6,222,675 putative TFBSs on the promoter regions of common bean genes. Out of the predicted sites, 219,742 unique sites were conserved between *P. vulgaris* and at least one of the studied species (Fig. 3A, Supplementary Table 1, Supplementary Table 2) We hypothesize that the drastic reduction of predicted sites following the conservation test reflects a high false positive rate of computational TFBS prediction software caused by the short and degenerate nature of binding motifs [42, 43], which is reduced upon application of the conservation requirement. Approximately 3.8% of the conserved motifs were present on the promoters of all four species, 29% were conserved between *P. vulgaris* and two other species, and 67% were conserved between common bean and only one of the studied species (Fig. 3B). More than 90% of all genes that exhibit similar promoters have at least one conserved motif and 16% of all identified conserved TFBSs were located within annotated upstream genes. The presence of regulatory elements in the gene body has been documented previously and is believed to be a part of the regulatory landscape in plants [8].

Finally, we compared the reference assembly v1.0 against the v2.1 as this is the newer version and is mostly used by the scientific community. About 98% of all conserved TFBSs identified in the reference assembly 1.0 are present in v2.1 within 20 nucleotides upstream or downstream the original coordinates. Supplementary Tables 1 and 2 list all conserved TFBSs within the 2000 nucleotides upstream the TSS of bean genes in reference genome v2.1 and v1.0 respectively.

The number of conserved TFBSs associated with each transcription factor (TF) family is depicted in Fig. 3C. The most commonly conserved sites corresponded to the ERF family, which is the largest subfamily of the plant specific AP2/ERF superfamily involved in development and stress response regulation. The common bean genome contains 95 ERF genes and is one of the largest families, and therefore the abundance of TFBSs from this family is expected. Other abundant TFBSs correspond to widely present transcription factor families such as MYB, bHLH, and C2H2. In contrast, the least represented TFBS motif is that of the highly specific pioneer TF family LFY, followed by EIL, SRS, and TALE. The density of conserved TFBSs along the length of the promoter closely mirrored that of similarity levels between promoters (Fig. 3D). The number of conserved TFBSs was maximal near the TSS and rapidly decreased in upstream positions (Fig. 3E).

To investigate the extent to which species retain unique sets of genes with conserved TFBSs, we conducted a GO functional enrichment analysis of genes that have conserved TFBSs in their promoters. Our analysis of *P. vulgaris* paired with each of the three species revealed that sets of genes with conserved sites exhibit diverse functional profiles across species (Supplementary Table 3). About 14% of all enriched GO terms were shared between common bean and all three species. This core set of GO terms primarily related to metabolism (regulation of cellular metabolic process, regulation of nitrogen compound metabolic process, regulation of nucleobase-containing compound metabolic process) and transcriptional regulation (regulation of nucleic acid-templated transcription, regulation of transcription, DNA-templated, transcription regulator activity).

Biological roles enriched within genes that have conserved TFBSs between the common bean and one of the other species tend to be more specific. For example, terms related to organ development were enriched in orthologous genes with conserved TFBSs between *P.vulgaris* and *V. angularis* (plant organ development, anatomical structure morphogenesis). *V. radiata* orthologous pairs were enriched in catabolism-related activities (carbon-carbon lyase activity, catabolic process, catalytic activity) as well as terms related to protein modification (cellular protein modification process, glycosylation). Additionally, some protein cellular organization and transport terms were shared between *V. angularis* and *G. max* orthologs (cytoskeletal protein binding, intracellular transport, localization, microtubule binding, etc). *G. max* orthologous pairs with conserved TFBSs were enriched in actin filament binding and organization, cell differentiation, and phosphatase activity-related functions (Supplementary Table 3).

### Conserved TFBSs are associated with accessible chromatin regions

Regulatory elements are expected to be situated in ACRs of the tissues where they are active to enable transcription factor binding. We investigated the association between ACR and our conserved TFBS detections using public ATAC-seq data of the bean leaf tissue [36].

Out of 19,089 reported ACRs, 6,721 were found within the promoters of 5,331 genes containing conserved motifs. This gene subset was highly enriched in photosynthesis and metabolism-related processes such as RNA, nitrogen, phosphorus, and hormone metabolic processes, as expected of leaf tissue. We found that 51.8% of unique conserved motifs of these genes were located within ACRs, while ACRs accounted for only 19% of the studied promoter length. The χ^2^ test showed a significant relationship between ACRs and the number of conserved nucleotides within them (*p* < 0.00001), revealing that our TFBS conservation analysis is consistent with features of chromatin accessibility and further validating our approach.

### Biological roles associated with TF families based on the representation of conserved motifs

We then asked if TF families with conserved TFBS were involved in the regulation of specific cellular functions by conducting GO enrichment analyses of their target genes. We found a total of 198 enriched GO terms associated with 38 TF families with most TF families showing between 10 and 30 enriched GO terms (Supplementary Table 4). Supplementary Fig. 2 shows the relationship between TF families and their associated enriched GO terms. Large TF families such as ERF, MYB, and C2H2 had significant association with numerous terms (35, 39, and 45, respectively), with some functions such as ‘regulation of transcription,’ ‘glycosyltransferase activity,’ ‘defense response,’ and ‘monooxygenase activity’ being shared among these three families. However, the number of significantly associated GO terms was not directly correlated with the number of conserved sites for a particular TF family. For instance, the MYB family has approximately half the number of potential conserved binding motifs as the ERF family, but it has ten more GO terms than ERF.

**Fig. 4:**
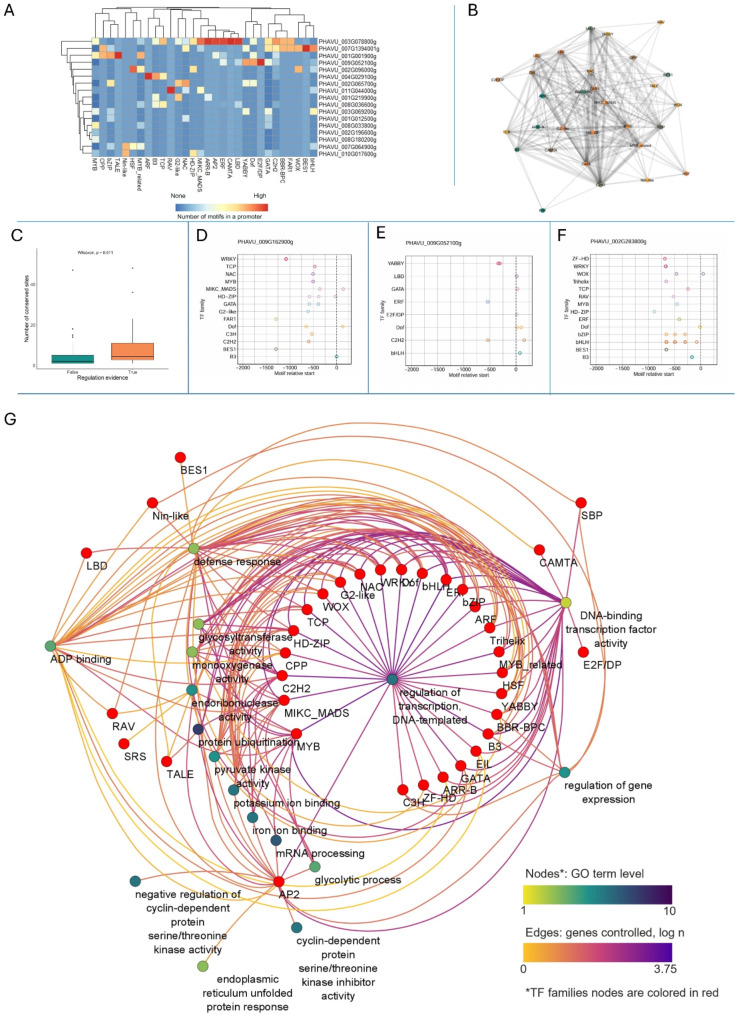
Literature-based analysis of regulatory potential of conserved motifs. **(A)** Representation of TF families on promoters of starch biosynthesis genes. Each row represents a gene, and each column represents motifs to a specific TF family. The color indicates the number of conserved TFBS in the promoter region. **(B)** Transcriptional network for bean starch biosynthesis genes inferred from our study. Nodes are TF and edge thickness represent the number of co-regulated genes. **(C)** The distribution of the number of conserved TFBSs by TF family by their literature evidence code on promoters of starch biosynthesis genes. The x-axis indicates the sum of conserved motifs to a TF family on promoters of all genes within a gene family. Evidence code 1 stands for no evidence of gene group being regulated by a TF family. Evidence code 2 indicates indirect evidence such as co-expression, and code 3 is assigned to interactions described plants. **(D)** Distribution of conserved motifs on the promoter region of *P. vulgaris SWEET10* gene (PHAVU_009G162900g). **(E)** Distribution of conserved motifs on the promoter region of *P. vulgaris* starch synthase I (PHAVU_009G052100g). The shaded area corresponds to the location of an upstream gene. **(F)** Distribution of conserved motifs on the promoter region of *P. vulgaris SWEET10* gene (PHAVU_002G283800g). **(G)** Network of significant GO terms associated with AP2 family and other TF families. GO term nodes are colored according to the term level, the edges are colored according to the number of genes that have conserved TFBSs to the corresponding TF family. TF nodes are colored in red

To further evaluate how our predictions are supported by current knowledge, we focused on the functions associated with the AP2 family. AP2 is a plant-specific TF family known to play critical roles in plant growth and development, including floral organ identity, leaf development, and response to environmental stresses [44, 45] In our analysis, we found 18 GO terms significantly associated with AP2 transcription factors (Fig. 4A) with multiple functions previously verified in other plant species. For instance, it is well-known that the AP2 transcription factor WRINKLED1 regulates pyruvate kinase (Pl-PKβ1) in *Arabidopsis* [46]. In our analysis, we determined that the AP2 family was significantly associated with the term ‘pyruvate kinase activity’ and had conserved binding motifs on the promoter of 13 genes with this biological function. Furthermore, AP2 transcription factors were associated with two terms related to cyclin-dependent protein kinases (CDK): ‘cyclin-dependent protein serine/threonine kinase inhibitor activity’ and ‘negative regulation of cyclin-dependent protein serine/threonine kinase activity’, which is consisted with reports demonstrating the involvement of this TF family and of CDKs in plant defense response mechanisms [47]. Additionally, AP2/ERF transcription factors interact with CDK8 during drought response in *Arabidopsis* [48]. Interestingly, no other TF families were identified to be associated with CDK regulation in our analysis (Fig. 4A).

Other biological roles associated with AP2 involved transcriptional regulation, consistent with their known role in complex regulatory cascades [49]. Also, AP2 was significantly associated with the ‘monooxygenase activity’ GO term, which is critical for plant growth and development. There is evidence that AP2 transcription factors bind to the promoter region of P450 monooxygenase in *Artemisia annua* [50]. Two more GO terms significantly associated with AP2 were ‘iron ion binding’ and ‘potassium ion binding’. Although we did not find evidence that AP2 TFs regulate iron ion binding genes directly, a number of AP2/ERF genes are Fe and Cu-responsive and act as repressors of Fe deficiency-responsive genes [51]. Moreover, the role of AP2 in potassium uptake in response to low-potassium conditions is well documented in *Arabidopsis* [52]. Lastly, the ‘glycolytic process’, ‘defense response’, and ‘endoplasmic reticulum unfolded protein response’ GO terms identified here were also previously shown to be regulated by the AP2 family [53, 54].

Overall, we confirmed 12 out of 18 significantly associated biological roles of the AP2 family through literature review. However, some of the AP2-associated GO terms, such as ‘endoribonuclease activity’, ‘mRNA processing’, ‘protein ubiquitination’, ‘ADP binding’, and two terms related to glycosyltransferase activity lacked literature support. While these results could be false discoveries, they may also indicate not yet reported functions of this family. For instance, AP2 transcription factor DREB2A activity has been shown to be mediated by E3 ubiquitin ligases in *Arabidopsis* [55]. The presence of AP2 binding motifs on promoters of genes involved in ubiquitination could suggest a potential self-regulatory feedback loop. Another example is the association of the AP2 family with genes involved in endoribonuclease activity, which is a component of plants’ immune response. While there is no direct evidence that AP2 is involved in regulating endoribonuclease activity, the AP2 transcription family is known to regulate plant defense systems, which could suggest a potential link.

In summary, this thorough examination of the literature supporting our predictions regarding the functional roles of conserved transcription factors in common bean indicates that our approach successfully aligns with established knowledge while also proposing plausible additional functional activities.

### Our approach is validated by experimental data and provides novel insights into the regulation of starch biosynthesis

Motivated by our interest in beans as staple food, we investigated if our approach could characterize the TF regulatory landscape of starch biosynthesis. Starch biosynthesis genes are essential for agricultural needs and are relatively well studied in multiple plant species. We selected starch-related gene families in the bean genome and studied conserved TFBS identified by our analysis. Out of the six gene groups involved in starch biosynthesis, which are represented by 29 genes according to the Plant Metabolic Network [56] 18 genes have conserved TFBS on their promoter regions (Fig. 4A). On average, each promoter region of starch biosynthesis genes contained 16 conserved motifs. Starch synthase III (SSIII), PHAVU_003G078800g, exhibited the highest number of conserved motifs, totaling 101 TFBSs. Although the regulation of this gene in the common bean has not been studied, starch synthase III is a pivotal enzyme in starch biosynthesis, responsible for elongating both amylose and amylopectin in the starch granule [57]. This observation aligns with the theory that core genes often possess a more conserved regulatory makeup [18]. The predominant conserved motifs identified in the promoter region of starch synthase III correspond to the ERF [36], MIKC MADS [10], and C2H2 [10] TF families. All three families have been implicated in starch biosynthesis across various plant species [58–61]. ERF TFs have previously been demonstrated to bind directly to the SSIII promoter [62], while MIKC MADS and C2H2 are recognized as regulators of starch biosynthesis and degradation, albeit without direct evidence of binding to the SSIII promoter.

To expand our sample size, we included the SWEET transporters paralogous group in the subset of starch biosynthesis genes, given its crucial role in seed starch filling throughout development [63]. In plants, the SWEET gene family comprises a group of membrane proteins that facilitate the transport of sucrose and other sugars between different plant tissues. Members of this family play important roles in seed development, phloem loading, and nectar secretion. Our aim was to explore the relationships within the same TF family on the promoters of this paralogous group by constructing a network of cooccurring families, where the edge weight indicates the number of cooccurrences (Fig. 4B). The network unveils that TF families such as C2H2 and MYB, bHLH, bZIP, TCP and Dof, among others, tend to cooccur multiple times on the promoters of SWEET genes. Conversely, HSF, YABBY, and Nin-like transcription factors exhibit a tendency to infrequently cooccur with other families in SWEET promoters.

Next, we performed a systematic literature search for experimental evidence supporting the regulation of these starch biosynthesis genes by the TF predicted by our approach (Supplementary Table 5). We observed that TF families with established regulatory roles tend to have numerous TFBSs on the promoters of their potential genes (Fig. 4C). We used the Kruskal-Wallis test to assess the significance of this observation. We detected a significant (adjusted *p* = 0.011) relationship between the number of conserved TFBSs associated with each gene family and the evidence supporting the regulation of the gene family by the predicted TF family. These results further validated our functional TF predictions.

There were 5 TF families with a high number (15 and more) of conserved motifs on the promoters of the same gene group for which literature support could not be found: MYB on promoters of starch glucanohydrolase, C2H2 on promoters of starch synthases, and B3, bHLH, HD-ZIP on promoters of SWEET genes. We consider these to be strong candidates for future experimental testing, particularly since some of these families have shown potential as active regulators. For example, Spies et al. (2022) showed that HD-ZIP transcription factors regulate *SWEET10* and *SWEET11* genes in *Arabidopsis* [64]. However, the authors did not identify potential binding sites to these transcription factors on the promoter of the studied genes. Notably, our analysis of common bean found that the promoters of SWEET genes have 15 conserved motifs for HD-ZIP. The *SWEET10* (PHAVU_009G162900g) promoter contains 6 conserved TFBSs for TF HD-ZIP located between − 579 to -10 upstream the TSS. The distribution of conserved motifs on the gene’s promoter is visualized in Fig. 4D.

Figure 4E shows another example of a gene (PHAVU_009G052100g, starch synthase I) with distal conserved motifs with potential regulatory roles. In *Z. maize*, ERF TF ZmEREB156 regulates starch biosynthesis by interacting with starch synthase *ZmSSIIIa* [62]. We identified 45 conserved binding motifs to ERF transcription factors in the promoter regions of common bean starch synthases. The promoter region of PHAVU_009G052100g contains a total of 11 conserved motifs to ERF TFs that are located approximately 500 base pairs upstream of the TSS.

The *SWEET10* (PHAVU_002G283800g) gene contains 10 clustered conserved TFBS for WRKY transcription factors. These TFBS are dispersed between − 700 and − 600 nucleotides relative to the TSS as shown in Fig. 4F. The presence of these conserved TFBS suggests that WRKY TFs play a regulatory role in the expression of this *SWEET* gene in the common bean. However, we were not able to identify proof that SWEET10 is regulated by WRKY TFs in other plant species. Nonetheless, it was shown that the activity of *MdSWEET9b* in apples is regulated by the WRKY transcription factor MdWRKY9 [65]. SWEET9 and SWEET10 belong to the cade III of SWEET transporters and share most of the functions [66], therefore these genes could be regulated by the same TFs.

### Conserved TFBSs have genetic variation across different common bean accessions

One of the unique qualities of common bean species is the presence of two distinct gene pools– Andean and Mesoamerican– that evolved independently. The Andean pool diverged from the Mesoamerican pool approximately 165,000 years ago, and both pools underwent domestication around 8,000 years ago, independently. One prominent difference between the two gene pools is their starch content. The seeds of the wild-type Andean accession are larger in mass and have a higher relative starch content compared to those of the Mesoamerican accession. The domestication process in both gene pools has resulted in selection for larger seeds, achieved through an increase in starch content. Consequently, both domesticated accessions exhibit higher starch content than their wild ancestors.

The independent evolution of the two gene pools provides a natural experiment to study how different genetic backgrounds influence the regulation of important traits. Investigating transcription factor binding site variability can reveal how evolutionary pressures have shaped the regulatory networks in each gene pool, offering a deeper understanding of plant adaptation and domestication processes.

**Fig. 5:**
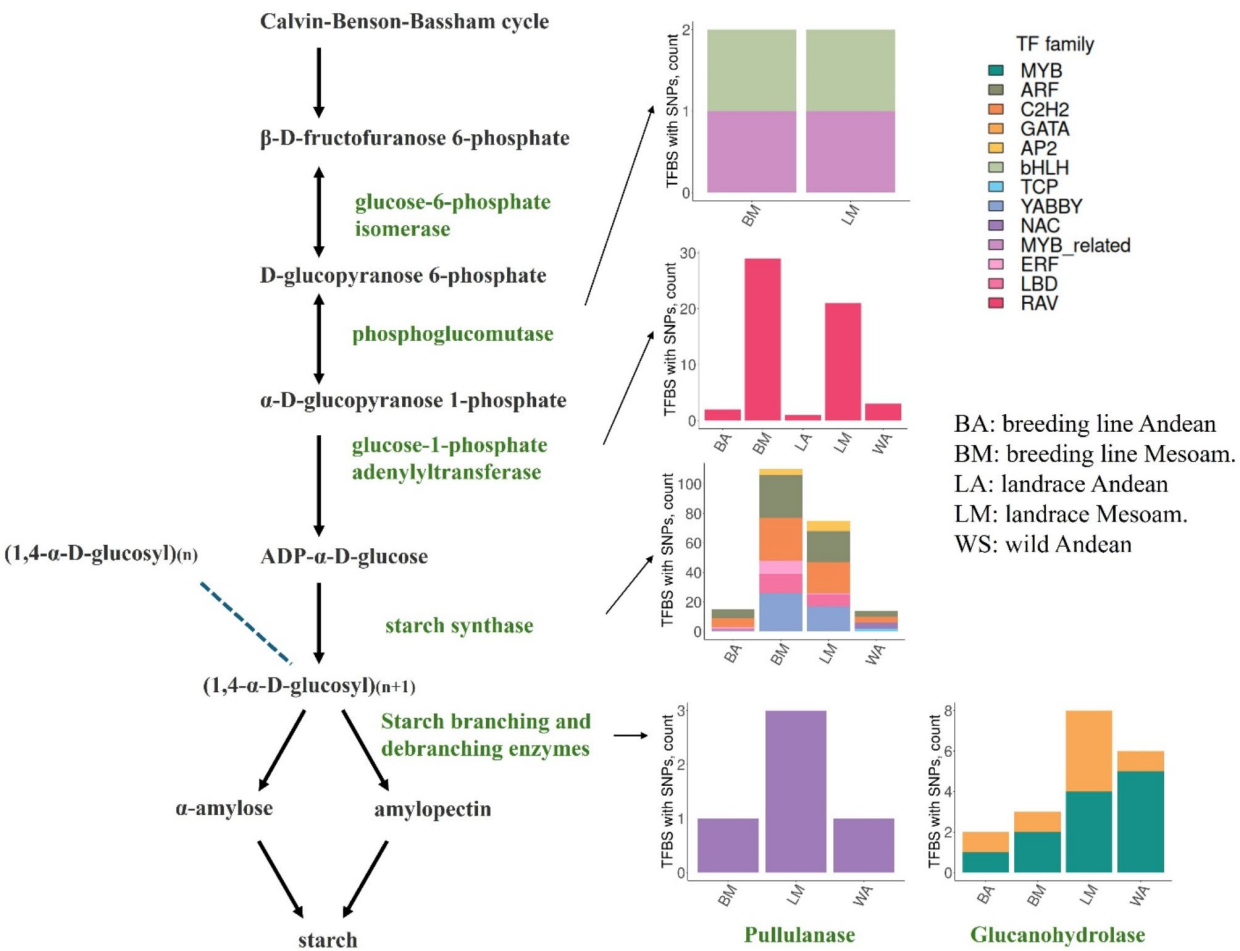
Genetic variants in conserved TFBSs of starch biosynthesis genes in the context of starch biosynthesis pathway

The analysis of TFBS polymorphisms between Andean and Mesoamerican accessions, as well as those arising from domestication within each gene pool, may provide valuable insights into the genetic mechanisms underlying the observed differences in starch content. To explore this issue, we examined the genome sequences of 125 accessions across six genotypic groups: breeding Andean [15], breeding Mesoamerican [3], cultivar Andean [19], cultivar Mesoamerican [26], landrace Andean [29], landrace Mesoamerican [21], and wild Andean [12]. Figure 5 presents the overall number of conserved TFBSs containing polymorphisms across all analyzed accessions. Out of the 20 tested starch genes, the promoters of 8 genes contained polymorphisms within predicted conserved TFBSs, including AGPase, 3 starch synthases, 2 glucanohydrolases, phosphoglucomutase, and pullulanase. In total, 16 polymorphisms were identified across all samples, affecting 20 conserved TFBSs. The TF families whose binding sites are impacted by these polymorphisms across different accessions include RAV, ARF, C2H2, YABBY, ERF, LBD, NAC, and AP2.

The bar plots represent the number of polymorphisms across all samples for various gene groups involved in different stages of the starch biosynthesis pathway, which are affected by genetic pool evolution. The gene groups include phosphoglucomutase, glucose-1-phosphate adenylyl transferase (AGPase), starch synthase, and two debranching enzymes (pullulanase and glucanohydrolase) and exhibit genetic variation within conserved transcription factor binding sites (TFBSs).

Since the reference genome used in this study was derived from the Andean landrace accession (G19833), only one landrace accession exhibited polymorphism within the conserved TFBS (Pvulgaris_442_v2.1:11.3960161 A > T). This SNP, located in the promoter of the glucose-1-phosphate adenylyltransferase (AGPase) gene, lies within a TFBS associated with the RAV transcription factor family (c.-200-193). Notably, all 50 Mesoamerican accessions shared this SNP, whereas only 6 out of 75 Andean accessions carried this variation. Although RAV transcription factors, a subgroup within the APATELA2 family, are known to regulate starch biosynthesis [44], a direct link between RAV and AGPase has not been previously reported. The high prevalence of this SNP in the Mesoamerican gene pool suggests it may contribute to the differences in starch biosynthesis between Andean and Mesoamerican accessions.

Another distinguishing genetic variation (Pvulgaris_442_v2.1:4.3458171 A > G) between the Andean and Mesoamerican accessions was located in the promoter of the starch synthase gene Phvul.004G029100, affecting TFBSs for ARF (c.-136-127) and C2H2 (c.-129-118) transcription factors. This SNP is present in 10 out of 74 Andean and all 50 Mesoamerican accessions. A third notable variation (Pvulgaris_442_v2.1:9.10085100_10085134delATATTGTAATAATAATTCTACGGATAAAAAGTAT), located in the promoter of the starch synthase gene Pvul.009G052100, was observed in 39 out of 50 Mesoamerican accessions compared to only 1 out of 75 Andean accessions. This deletion affects a conserved TFBS for YABBY (c.-340-331) transcription factors. To date, there is no evidence suggesting that ARF, C2H2, or YABBY transcription factors regulate the expression of starch synthase genes. Additionally, we did not observe any significant genetic variation in conserved TFBSs between wild and domesticated accessions.

A complete list of SNPs is provided in Supplementary Table 6.

Our findings suggest that specific genetic variations, particularly those prevalent in the Mesoamerican gene pool, may play a significant role in starch biosynthesis regulation, potentially influencing the distinct starch content observed between the two gene pools. However, no major TFBS polymorphisms were detected between wild and domesticated accessions, indicating that these regulatory changes are more closely associated with the divergence between the Andean and Mesoamerican gene pools rather than domestication alone.

## Discussion

This study addressed the identification of conserved transcription factor binding sites (TFBS) in *P. vulgaris* employing a comparative genomics approach. Using this approach, we annotated a total of 219,742 TFBS in 12,632 *P. vulgaris* genes; approximately 43% of the annotated genes represent an unprecedented source of potential gene regulatory data for the common bean. This data set can be queried by inputting a user-provided list of bean genes with code provided in this study (https://github.com/ConesaLab/beanTFBSs). The output consists of a TF regulatory report that includes TFBS, promoter maps, heatmaps with conserved motif frequencies and TF co-occurrence networks (illustrated in Fig. 4A-F). The occurrence of each TF family was assessed against the entire genome occurrence using a binomial test. This information supports the generation and testing of hypotheses regarding the relationship between a gene and a TF family, offering insights into possible false positives.

Our findings underscore the functional significance of conserved TFBSs, which are in line with preceding studies that explored the TFBS conservation across evolutionarily related plant species [17]. By leveraging orthologous genes of *V. angularis*, *V. radiata*, and *G. max* as a reference set, we predicted a regulatory landscape governing gene expression in *P. vulgaris*. We investigated regulatory programs related to functions such as nutrient transport and biosynthesis, which demonstrated that our approach effectively assigned regulatory information to key processes essential for plant health and productivity.

The strengths of our approach lie in the scalability and efficiency of computational comparative genomics. The identification and pairing of conserved TFBS to their cognate TF in crops with limited genetic resources is an effective strategy to expand their genetic characterization. However, we recognize the risks of detecting false positives and negatives with this approach. False negatives arise from the species-specific nature of some regulatory elements, which, by definition, do not exhibit conservation across related species and therefore are not captured by our method. Additionally, restricted promoter span hinders the ability of the approach to capture distal and intragenic TFBS, which are known to be part of plants’ regulatory landscape [67]. False positive may occur due to the redundancy and low specificity of some transcription factor binding motifs.

Despite these risks, we show that our results faithfully recapitulate the extant plant regulatory knowledge as indicated in the current scientific literature for the AP2 family of transcription factors and the starch biosynthesis genes. Altogether, these observations support the reliability of our approach and highlight the potential of comparative genomics to predict plausible regulatory elements in non-model species.

We also made some observations based on our findings. For instance, we detected genes with conserved coding and promoter sequences which were associated with metabolism, that of nitrogen in particular which is unique among legumes and distinguishes them from other taxa. At the same time, the unique commonalities detected between the different pairings of *P. vulgaris* with each of the other three species point at the areas of divergent evolution that has taken place among them. Furthermore, we also identified potential candidates for experimental testing and highlighted the importance of analyzing distally located conserved motifs, which could have regulatory potential. In essence, our findings not only illuminate specific facets of gene regulation but also affirm the utility of employing comparative genomics as a strategic tool for hypothesis generation and testing, particularly in contexts where informational constraints persist within non-model organisms.

Polymorphism analysis highlighted significant variations in TFBS regions between Andean and Mesoamerican accessions that may contribute to the observed differences in starch content between these gene pools. The identification of genetic variation within the promoters of key starch biosynthesis genes, such as those for AGPase and starch synthase, and their association with transcription factors like RAV, ARF, C2H2, and YABBY, suggests potential regulatory differences in starch metabolism between Andean and Mesoamerican accessions. The fact that these variations are prevalent in the Mesoamerican accessions, yet relatively rare in the Andean accessions, underscores the possibility that these polymorphisms may play a role in the adaptation and domestication processes specific to each gene pool. Moreover, the lack of significant TFBS polymorphisms between wild and domesticated accessions suggests that these regulatory changes might be more closely associated with the divergence of the Andean and Mesoamerican gene pools rather than with domestication per se. In addition, this observation also suggests that domestication may have targeted coding DNA sequences more frequently. Further functional studies are needed to elucidate the direct impact of these polymorphisms on gene expression and starch biosynthesis, which could provide deeper insights into the genetic basis of starch content variation in common bean.

The motivation of this work arose from the necessity to improve genome annotation in the common bean to support genetic improvement programs that leverage the potential of this crop to address current food security threats. In summary, our research advances the field by providing a comprehensive analysis of conserved TFBS in *P. vulgaris*, providing a valuable resource to study regulatory networks governing gene expression in this important crop. While we specifically discuss the regulatory program involved in starch biosynthesis, we expect that our resource will provide insights into the regulation of other metabolic and developmental processes relevant to bean production. Finally, our comparative genomics approach could be easily extended to other crop species that face similar annotation challenges as the common bean.

## Conclusions

This study presents a comprehensive analysis of the conservation and potential functional roles of transcription factor binding sites across the promoters of common bean genes. Our findings highlight the evolutionary constraints on promoter regions, particularly those near transcription start sites. Our analysis revealed that highly conserved genes are likely to have conserved promoter regions, further supporting the idea that regulatory regions are subject to purifying selection across species. The identification of highly abundant conserved TFBSs, particularly those associated with key transcription factor families such as ERF, MYB, and C2H2, underscores their importance in regulatory networks governing essential biological processes like stress response, metabolism, and development.

The study also uncovered the presence of conserved TFBSs within accessible chromatin regions (ACRs), reinforcing the notion that these sites are functionally relevant and likely contribute to gene regulation in a tissue-specific manner. Furthermore, our investigation into the starch biosynthesis pathway, a critical agricultural trait in common beans, demonstrated the potential of this approach to hypothesize novel regulatory interactions. The identification of conserved TFBSs in the promoters of starch biosynthesis genes, along with the cross-reference validation of known regulatory relationships, highlights the utility of this approach in predicting regulatory elements in crop species.

The genetic variation in conserved TFBSs between different common bean accessions offers insights into the evolutionary and domestication processes that have shaped the regulatory landscapes of these gene pools. The differences in starch content between the Andean and Mesoamerican gene pools, possibly influenced by variation in conserved TFBSs, provide a basis for future studies aimed at improving crop traits through targeted breeding.

It is important to note that computational predictions are prone to both, false positive and false negative results. When referring to the result of this work, the reader ought to keep in mind that species-specific TFBSs cannot be captured by the conservation test. Additionally, the presence of a conserved TFBS sequence in two or more evolutionary closely related species suggests but not confirms the presence of an active binding site. Literature-based validation of our predictions supports their robustness; however, specific experimental validations, which are beyond the scope of this work, would be necessary to confirm additional findings.

In conclusion, this study advances our understanding of the conservation and function of regulatory elements in common bean and related species, offering valuable insights into the evolution of gene regulation in legumes. The results open new avenues for exploring the regulatory mechanisms underlying important agricultural traits.

## Electronic supplementary material

Below is the link to the electronic supplementary material.

Supplementary Material 1: Supplementary figures 1-2

Supplementary Material 2: Supplementary Table 1. List of conserved motifs on P. vulgaris v2.1 promoters.

Supplementary Material 3: Supplementary Table 2. List of conserved motifs on P. vulgaris v1.0 promoters.

Supplementary Material 4: Supplementary Table 2. Functional enrichment analysis by species.

Supplementary Material 5: Supplementary Table 3. Enriched functions within genes with conserved motifs by transcription factor families.

Supplementary Material 6: Supplementary Table 4. Literature-based support for starch genes.

Supplementary Material 7: Supplementary Table 5. Genetic variants in starch biosynthesis genes.

## Data Availability

Public DNA sequences used in this study: NCBI, PRJNA471678P. *P. vulgaris* starch genes promoter region genetic variation: 10.6084/m9.figshare.28270868.v1. The rest of tables generated within this study are provided within supplementary information files. The datasets analyzed and corresponding scripts during the current study are available in https://github.com/ConesaLab/beanTFBSs.

## Abbreviations

ACR: Accessible Chromatin Region
GO: Gene Ontology
GOC: Gene Order Conservation
TF: Transcription Factor
TFBS: Transcription Factor Binding Site
TSS: Transcription Start Site
WGA: Whole Genome Alignment
