# Supplementary material for: Profiling conserved transcription factor binding motifs in Phaseolus vulgaris through comparative genomics
Source: BMC Genomics. 2025 Feb 20;26:169. doi: 10.1186/s12864-025-11309-2 (PMC11841308; doi:10.1186/s12864-025-11309-2)
Supplement: Supplementary file 1 — Supplementary Material 1: Supplementary figures 1-2 [file 12864_2025_11309_MOESM1_ESM.pdf]

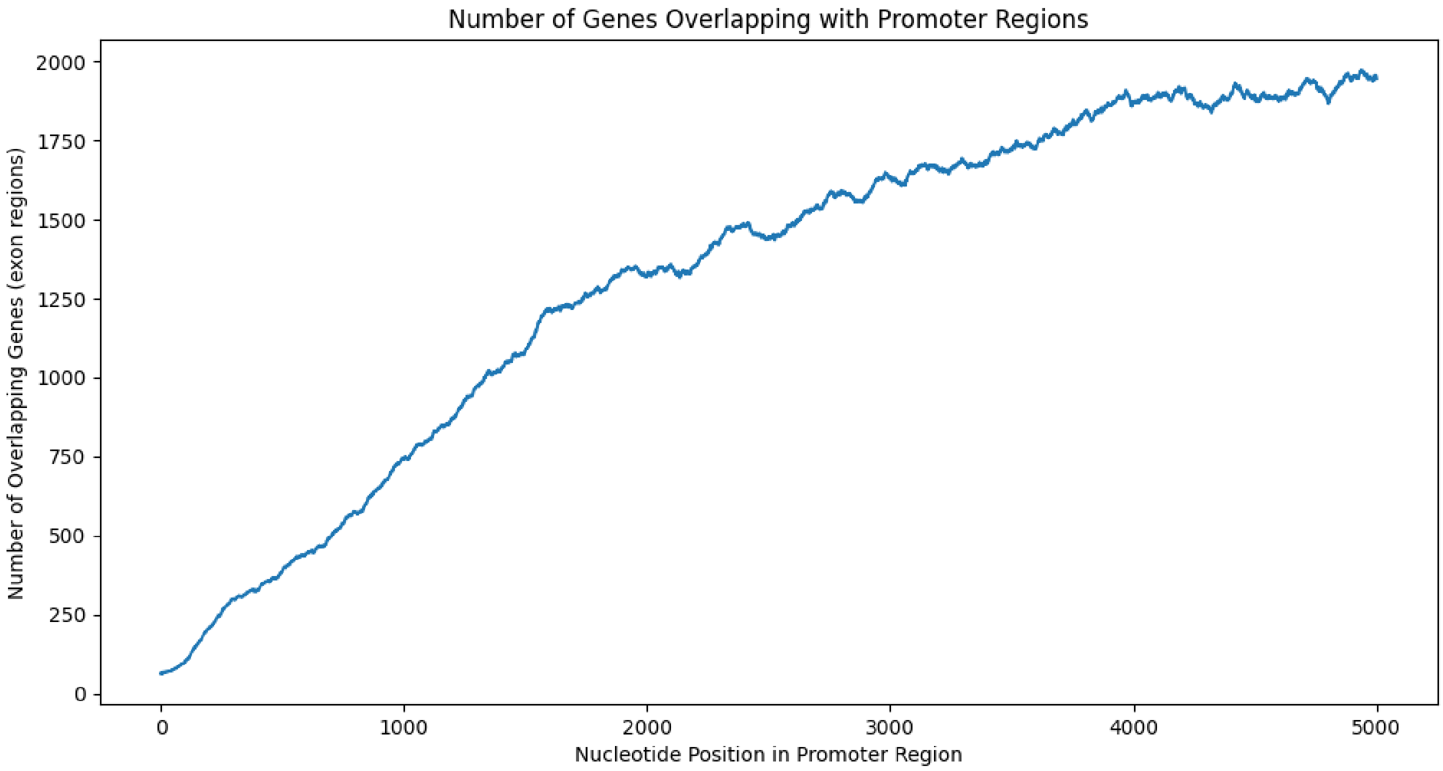

Supplementary Figure 1. Presence of coding sequence of upstream genes on the analyzed promoters of *P. vulgaris*.

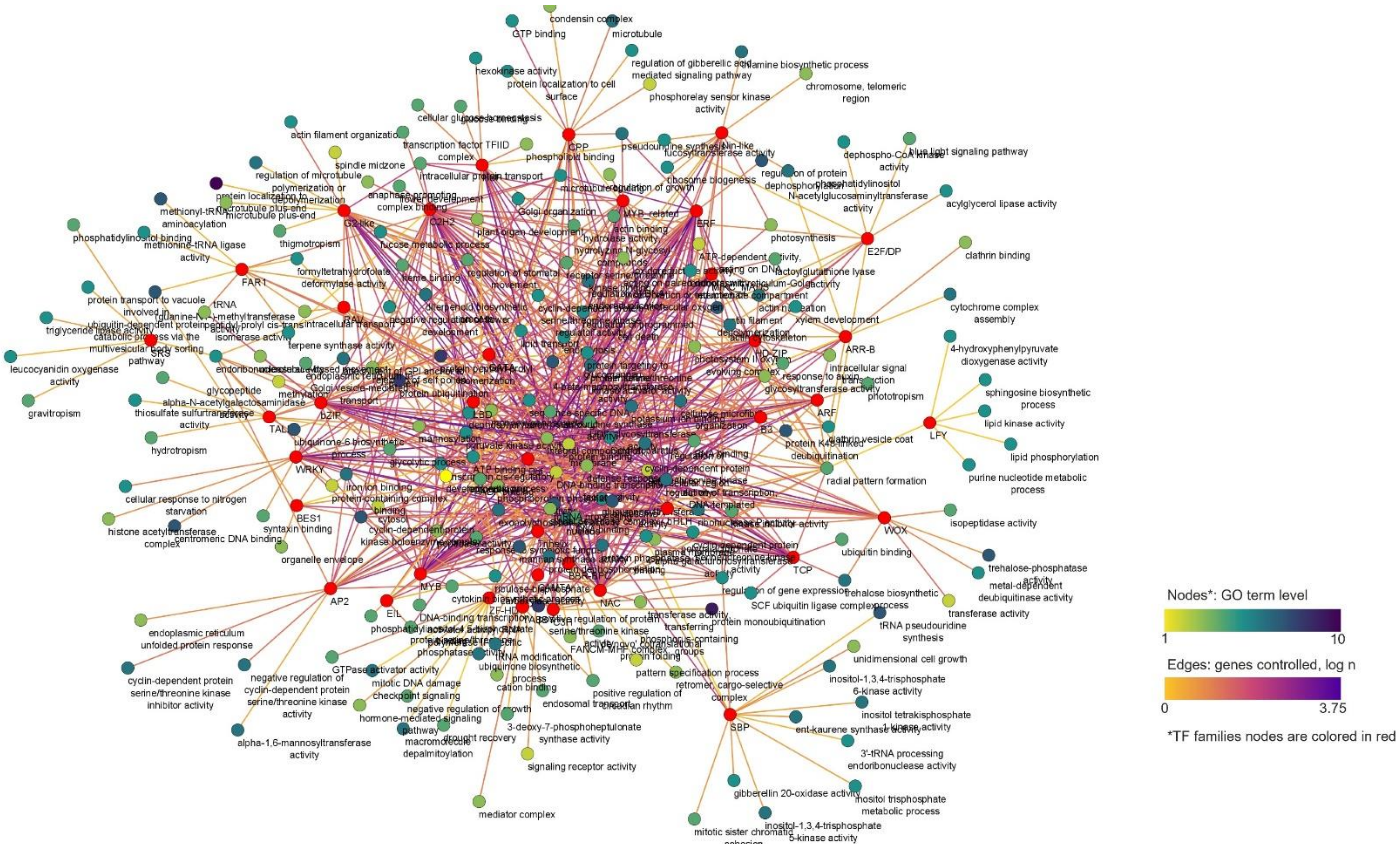

Supplementary Figure 2. Transcription factor families (red nodes) and their associated gene ontology terms based on functionality of genes with conserved motifs to each of the families.
