# Supplementary material for: Profiling conserved transcription factor binding motifs in Phaseolus vulgaris through comparative genomics
Source: BMC Genomics. 2025 Feb 20;26:169. doi: 10.1186/s12864-025-11309-2 (PMC11841308; doi:10.1186/s12864-025-11309-2)
Supplement: Supplementary file 6 — Supplementary Material 6: Supplementary Table 4. Literature-based support for starch genes. [file 12864_2025_11309_MOESM6_ESM.docx]

Supplemental Table 1. Literature analysis of conserved sites identified on promoters of some genes involved in starch metabolism.

|  | Gene group | **TF family** | **Regulation evidence** | **Number of conserved sites** | **Evidence** |
| --- | --- | --- | --- | --- | --- |
| 1 | glucose-1-phosphate adenylyltransferase | **G2-like** | False | 4 | NA |
| 2 | glucose-1-phosphate adenylyltransferase | **B3** | False | 1 | NA |
| 3 | glucose-1-phosphate adenylyltransferase | **bHLH** | True | 2 | (Bian et al., 2021; Feng et al., 2018) |
| 4 | glucose-1-phosphate adenylyltransferase | **NAC** | True | 6 | (J. Wang et al., 2020)(Z. Zhang et al., 2019) |
| 5 | glucose-1-phosphate adenylyltransferase | **MIKC_MADS** | False | 5 | NA |
| 6 | **glucose-1-phosphate adenylyltransferase** | **bZIP** | True | 4 | (Song et al., 2020)(Chen et al., 2016a; J.-C. Wang et al., 2013) |
| 7 | glucose-1-phosphate adenylyltransferase | **MYB** | True | 1 | (Y. Zhang et al., 2020)(Luo et al., 2021; NILSSON et al., 2007; C. Wang et al., 2023) |
| 8 | glucose-1-phosphate adenylyltransferase | **ARR-B** | False | 1 | NA |
| 9 | glucose-1-phosphate adenylyltransferase | **NAC** | True | 5 | (J. Wang et al., 2020; Z. Zhang et al., 2019) |
| 10 | glucose-1-phosphate adenylyltransferase | **Dof** | True | 1 | (Gaur et al., 2011)(J. Wu et al., 2019a) |
| 11 | glucose-1-phosphate adenylyltransferase | **YABBY** | False | 1 | NA |
| `q | glucose-1-phosphate adenylyltransferase | **GATA** | False | 1 | NA |
| 13 | glucose-1-phosphate adenylyltransferase | **RAV** | False | 2 | NA |
| 14 | glucose-6-phosphate isomerase | **MYB** | False | 4 | NA |
| 15 | phosphoglucomutase | **bHLH** | False | 2 | NA |
| 16 | phosphoglucomutase | **MYB** | False | 1 | NA |
| 17 | phosphoglucomutase | **HD-ZIP** | False | 1 | NA |
| 18 | phosphoglucomutase | **MYB_related** | False | 3 | NA |
| 19 | phosphoglucomutase | **C2H2** | False | 1 | NA |
| 20 | phosphoglucomutase | **HSF** | False | 2 | NA |
| 21 | phosphoglucomutase | **WOX** | False | 1 | NA |
| 22 | pullulanase | **BBR-BPC** | False | 1 | NA |
| 23 | pullulanase | **BES1** | False | 3 | NA |
| 24 | pullulanase | **bHLH** | False | 8 | NA |
| 25 | pullulanase | **bZIP** | False | 1 | NA |
| 26 | pullulanase | **C2H2** | False | 7 | NA |
| 27 | pullulanase | **CPP** | False | 1 | NA |
| 28 | pullulanase | **FAR1** | False | 1 | NA |
| 29 | pullulanase | **GATA** | False | 3 | NA |
| 30 | pullulanase | **MIKC_MADS** | False | 2 | NA |
| 31 | pullulanase | **NAC** | False | 2 | NA |
| 32 | pullulanase | **WOX** | False | 1 | NA |
| 33 | pullulanase | **YABBY** | False | 1 | NA |
| 34 | starch glucanohydrolase | **ERF** | False | 5 | NA |
| 35 | starch glucanohydrolase | **bHLH** | True | 5 | (Bello et al., 2019) |
| 36 | starch glucanohydrolase | **MIKC_MADS** | False | 2 | NA |
| 37 | starch glucanohydrolase | **bZIP** | False | 5 | NA |
| 38 | starch glucanohydrolase | **MYB** | False | 18 | NA |
| 39 | starch glucanohydrolase | **HD-ZIP** | False | 1 | NA |
| 40 | starch glucanohydrolase | **MYB_related** | False | 5 | NA |
| 41 | starch glucanohydrolase | **GATA** | False | 4 | NA |
| 42 | starch glucanohydrolase | **TALE** | False | 1 | NA |
| 43 | starch glucanohydrolase | **AP2** | False | 1 | NA |
| 44 | starch glucanohydrolase | **C2H2** | False | 4 | NA |
| 45 | starch glucanohydrolase | **CPP** | False | 1 | NA |
| 46 | starch glucanohydrolase | **Dof** | False | 1 | NA |
| 47 | starch glucanohydrolase | **Nin-like** | False | 1 | NA |
| 48 | starch glucanohydrolase | **YABBY** | False | 1 | NA |
| 49 | starch glucanohydrolase | **ARR-B** | False | 4 | NA |
| 50 | starch glucanohydrolase | **BBR-BPC** | False | 1 | NA |
| 51 | starch glucanohydrolase | **CAMTA** | False | 2 | NA |
| 52 | starch synthase | **ARF** | False | 3 | NA |
| 53 | starch synthase | **ERF** | True | 48 | (Huang et al., 2016) |
| 54 | starch synthase | **G2-like** | False | 1 | NA |
| 55 | starch synthase | **B3** | False | 4 | NA |
| 56 | starch synthase | **bHLH** | True | 3 | (Liu et al., 2023) |
| 57 | starch synthase | **NAC** | True | 2 | (Xiao et al., 2021) |
| 58 | starch synthase | **MIKC_MADS** | False | 10 | NA |
| 58 | starch synthase | **TCP** | False | 7 | NA |
| 60 | starch synthase | **bZIP** | True | 1 | (Chen et al., 2016b; Q. Dong et al., 2019; J.-C. Wang et al., 2013) |
| 61 | starch synthase | **MYB** | True | 8 | (Hu et al., 2021) |
| 62 | starch synthase | **HSF** | False | 1 | NA |
| 63 | starch synthase | **LBD** | False | 8 | NA |
| 64 | starch synthase | **Dof** | True | 3 | (Gaur et al., 2011)(Qi et al., 2017; J. Wu et al., 2019b) |
| 65 | starch synthase | **HD-ZIP** | False | 1 | NA |
| 66 | starch synthase | **MYB_related** | False | 4 | NA |
| 67 | starch synthase | **C2H2** | False | 14 | NA |
| 68 | starch synthase | **YABBY** | False | 2 | NA |
| 69 | starch synthase | **GATA** | False | 5 | NA |
| 70 | starch synthase | **E2F/DP** | False | 1 | NA |
| 71 | starch synthase | **FAR1** | False | 1 | NA |
| 72 | starch synthase | **Nin-like** | False | 1 | NA |
| 73 | sweet | **ARF** | False | 5 | NA |
| 74 | sweet | **ERF** | True | 23 | (Phukan et al., 2018) |
| 75 | sweet | **BES1** | False | 6 | NA |
| 76 | sweet | **G2-like** | False | 4 | NA |
| 77 | sweet | **B3** | False | 18 | NA |
| 78 | sweet | **AP2** | True | 6 | (Phukan et al., 2018) |
| 79 | sweet | **bHLH** | False | 47 | NA |
| 80 | sweet | **NAC** | True | 5 | (J. Wang et al., 2021)(Ren et al., 2021) |
| 81 | sweet | **WRKY** | True | 14 | (Yang et al., 2022) |
| 82 | sweet | **MIKC_MADS** | False | 11 | NA |
| 83 | sweet | **TCP** | True | 10 | (Z. Dong et al., 2019) |
| 84 | sweet | **bZIP** | True | 18 | (Mathan et al., 2021) |
| 85 | sweet | **MYB** | True | 36 | (Sun et al., 2019)(Li et al., 2022) |
| 86 | sweet | **HSF** | False | 1 | NA |
| 87 | sweet | **C3H** | False | 1 | NA |
| 88 | sweet | **Dof** | True | 6 | (Y. Wu et al., 2018) |
| 89 | sweet | **ARR-B** | False | 2 | NA |
| 90 | sweet | **Trihelix** | False | 1 | NA |
| 91 | sweet | **HD-ZIP** | False | 15 | (Spies et al., 2022) |
| 92 | sweet | **ZF-HD** | False | 1 | NA |
| 93 | sweet | **WOX** | False | 6 | NA |
| 94 | sweet | **C2H2** | False | 7 | NA |
| 95 | sweet | **YABBY** | False | 1 | NA |
| 96 | sweet | **GATA** | False | 4 | NA |
| 97 | sweet | **RAV** | False | 1 | NA |
| 98 | sweet | **E2F/DP** | False | 2 | NA |
| 99 | sweet | **FAR1** | False | 2 | NA |
| 100 | sweet | **BBR-BPC** | False | 4 | NA |
| 101 | sweet | **Nin-like** | False | 1 | NA |

**References**

Bello, B. K., Hou, Y., Zhao, J., Jiao, G., Wu, Y., Li, Z., Wang, Y., Tong, X., Wang, W., Yuan, W., Wei, X., & Zhang, J. (2019). NF ‐ YB1‐ YC12‐ bHLH 144 complex directly activates *Wx* to regulate grain quality in rice ( *Oryza sativa* L.). *Plant Biotechnology Journal*, *17*(7), 1222–1235. https://doi.org/10.1111/pbi.13048

Bian, S., Tian, T., Ding, Y., Yan, N., Wang, C., Fang, N., Liu, Y., Zhang, Z., & Zhang, H. (2021). bHLH Transcription Factor NtMYC2a Regulates Carbohydrate Metabolism during the Pollen Development of Tobacco (Nicotiana tabacum L. cv. TN90). *Plants*, *11*(1), 17. https://doi.org/10.3390/plants11010017

Chen, J., Yi, Q., Cao, Y., Wei, B., Zheng, L., Xiao, Q., Xie, Y., Gu, Y., Li, Y., Huang, H., Wang, Y., Hou, X., Long, T., Zhang, J., Liu, H., Liu, Y., Yu, G., & Huang, Y. (2016a). ZmbZIP91 regulates expression of starch synthesis-related genes by binding to ACTCAT elements in their promoters. *Journal of Experimental Botany*, *67*(5), 1327–1338. https://doi.org/10.1093/jxb/erv527

Chen, J., Yi, Q., Cao, Y., Wei, B., Zheng, L., Xiao, Q., Xie, Y., Gu, Y., Li, Y., Huang, H., Wang, Y., Hou, X., Long, T., Zhang, J., Liu, H., Liu, Y., Yu, G., & Huang, Y. (2016b). ZmbZIP91 regulates expression of starch synthesis-related genes by binding to ACTCAT elements in their promoters. *Journal of Experimental Botany*, *67*(5), 1327–1338. https://doi.org/10.1093/jxb/erv527

Dong, Q., Xu, Q., Kong, J., Peng, X., Zhou, W., Chen, L., Wu, J., Xiang, Y., Jiang, H., & Cheng, B. (2019). Overexpression of ZmbZIP22 gene alters endosperm starch content and composition in maize and rice. *Plant Science*, *283*, 407–415. https://doi.org/10.1016/j.plantsci.2019.03.001

Dong, Z., Xiao, Y., Govindarajulu, R., Feil, R., Siddoway, M. L., Nielsen, T., Lunn, J. E., Hawkins, J., Whipple, C., & Chuck, G. (2019). The regulatory landscape of a core maize domestication module controlling bud dormancy and growth repression. *Nature Communications*, *10*(1), 3810. https://doi.org/10.1038/s41467-019-11774-w

Feng, F., Qi, W., Lv, Y., Yan, S., Xu, L., Yang, W., Yuan, Y., Chen, Y., Zhao, H., & Song, R. (2018). OPAQUE11 Is a Central Hub of the Regulatory Network for Maize Endosperm Development and Nutrient Metabolism. *The Plant Cell*, *30*(2), 375–396. https://doi.org/10.1105/tpc.17.00616

Gaur, V. S., Singh, U. S., & Kumar, A. (2011). Transcriptional profiling and in silico analysis of Dof transcription factor gene family for understanding their regulation during seed development of rice Oryza sativa L. *Molecular Biology Reports*, *38*(4), 2827–2848. https://doi.org/10.1007/s11033-010-0429-z

Hu, Y., Li, Y., Weng, J., Liu, H., Yu, G., Liu, Y., Xiao, Q., Huang, H., Wang, Y., wei, B., Cao, Y., Xie, Y., Long, T., Li, H., Zhang, J., Li, X., & Huang, Y. (2021). Coordinated regulation of starch synthesis in maize endosperm by microRNAs and DNA methylation. *The Plant Journal*, *105*(1), 108–123. https://doi.org/10.1111/tpj.15043

Huang, H., Xie, S., Xiao, Q., Wei, B., Zheng, L., Wang, Y., Cao, Y., Zhang, X., Long, T., Li, Y., Hu, Y., Yu, G., Liu, H., Liu, Y., Huang, Z., Zhang, J., & Huang, Y. (2016). Sucrose and ABA regulate starch biosynthesis in maize through a novel transcription factor, ZmEREB156. *Scientific Reports*, *6*(1), 27590. https://doi.org/10.1038/srep27590

Li, D., Liu, B., Wang, Z., Li, X., Sun, S., Ma, C., Wang, L., & Wang, S. (2022). Sugar accumulation may be regulated by a transcriptional cascade of ABA-VvGRIP55-VvMYB15-VvSWEET15 in grape berries under root restriction. *Plant Science*, *322*, 111288. https://doi.org/10.1016/j.plantsci.2022.111288

Liu, Y., Xi, W., Wang, X., Li, H., Liu, H., Li, T., Hou, J., Liu, X., Hao, C., & Zhang, X. (2023). TabHLH95-TaNF-YB1 module promotes grain starch synthesis in bread wheat. *Journal of Genetics and Genomics*, *50*(11), 883–894. https://doi.org/10.1016/j.jgg.2023.04.002

Luo, G., Shen, L., Song, Y., Yu, K., Ji, J., Zhang, C., Yang, W., Li, X., Sun, J., Zhan, K., Cui, D., Wang, Y., Gao, C., Liu, D., & Zhang, A. (2021). The MYB family transcription factor *TuODORANT1* from *Triticum urartu* and the homolog *TaODORANT1* from *Triticum aestivum* inhibit seed storage protein synthesis in wheat. *Plant Biotechnology Journal*, *19*(9), 1863–1877. https://doi.org/10.1111/pbi.13604

Mathan, J., Singh, A., & Ranjan, A. (2021). Sucrose transport in response to drought and salt stress involves ABA‐mediated induction of *OsSWEET13* and *OsSWEET15* in rice. *Physiologia Plantarum*, *171*(4), 620–637. https://doi.org/10.1111/ppl.13210

NILSSON, L., MÜLLER, R., & NIELSEN, T. H. (2007). Increased expression of the MYB-related transcription factor, PHR1, leads to enhanced phosphate uptake in Arabidopsis thaliana. *Plant, Cell & Environment*, *30*(12), 1499–1512. https://doi.org/10.1111/j.1365-3040.2007.01734.x

Phukan, U. J., Jeena, G. S., Tripathi, V., & Shukla, R. K. (2018). MaRAP2-4, a waterlogging-responsive ERF from *Mentha,* regulates bidirectional sugar transporter *AtSWEET10* to modulate stress response in *Arabidopsis*. *Plant Biotechnology Journal*, *16*(1), 221–233. https://doi.org/10.1111/pbi.12762

Qi, X., Li, S., Zhu, Y., Zhao, Q., Zhu, D., & Yu, J. (2017). ZmDof3, a maize endosperm-specific Dof protein gene, regulates starch accumulation and aleurone development in maize endosperm. *Plant Molecular Biology*, *93*(1–2), 7–20. https://doi.org/10.1007/s11103-016-0543-y

Ren, Y., Huang, Z., Jiang, H., Wang, Z., Wu, F., Xiong, Y., & Yao, J. (2021). A heat stress responsive NAC transcription factor heterodimer plays key roles in rice grain filling. *Journal of Experimental Botany*, *72*(8), 2947–2964. https://doi.org/10.1093/jxb/erab027

Song, Y., Luo, G., Shen, L., Yu, K., Yang, W., Li, X., Sun, J., Zhan, K., Cui, D., Liu, D., & Zhang, A. (2020). *TubZIP28* , a novel bZIP family transcription factor from *Triticum urartu* , and *TabZIP28* , its homologue from *Triticum aestivum* , enhance starch synthesis in wheat. *New Phytologist*, *226*(5), 1384–1398. https://doi.org/10.1111/nph.16435

Spies, F. P., Raineri, J., Miguel, V. N., Cho, Y., Hong, J. C., & Chan, R. L. (2022). The Arabidopsis transcription factors AtPHL1 and AtHB23 act together promoting carbohydrate transport from pedicel-silique nodes to seeds. *Plant Science*, *315*, 111133. https://doi.org/10.1016/j.plantsci.2021.111133

Sun, W., Gao, Z., Wang, J., Huang, Y., Chen, Y., Li, J., Lv, M., Wang, J., Luo, M., & Zuo, K. (2019). Cotton fiber elongation requires the transcription factor Gh <scp>MYB</scp> 212 to regulate sucrose transportation into expanding fibers. *New Phytologist*, *222*(2), 864–881. https://doi.org/10.1111/nph.15620

Wang, C., Jiang, H., Gao, G., Yang, F., Guan, J., & Qi, H. (2023). CmMYB44 might interact with CmAPS2-2 to regulate starch metabolism in oriental melon fruit. *Plant Physiology and Biochemistry*, *196*, 361–369. https://doi.org/10.1016/j.plaphy.2023.01.047

Wang, J., Chen, Z., Zhang, Q., Meng, S., & Wei, C. (2020). The NAC Transcription Factors OsNAC20 and OsNAC26 Regulate Starch and Storage Protein Synthesis. *Plant Physiology*, *184*(4), 1775–1791. https://doi.org/10.1104/pp.20.00984

Wang, J., Wang, Y., Zhang, J., Ren, Y., Li, M., Tian, S., Yu, Y., Zuo, Y., Gong, G., Zhang, H., Guo, S., & Xu, Y. (2021). Correction: The NAC transcription factor *ClNAC68* positively regulates sugar content and seed development in watermelon by repressing *ClINV* and *ClGH3.6*. *Horticulture Research*, *8*. https://doi.org/10.1038/s41438-021-00710-z

Wang, J.-C., Xu, H., Zhu, Y., Liu, Q.-Q., & Cai, X.-L. (2013). OsbZIP58, a basic leucine zipper transcription factor, regulates starch biosynthesis in rice endosperm. *Journal of Experimental Botany*, *64*(11), 3453–3466. https://doi.org/10.1093/jxb/ert187

Wu, J., Chen, L., Chen, M., Zhou, W., Dong, Q., Jiang, H., & Cheng, B. (2019a). The DOF-Domain Transcription Factor ZmDOF36 Positively Regulates Starch Synthesis in Transgenic Maize. *Frontiers in Plant Science*, *10*. https://doi.org/10.3389/fpls.2019.00465

Wu, J., Chen, L., Chen, M., Zhou, W., Dong, Q., Jiang, H., & Cheng, B. (2019b). The DOF-Domain Transcription Factor ZmDOF36 Positively Regulates Starch Synthesis in Transgenic Maize. *Frontiers in Plant Science*, *10*. https://doi.org/10.3389/fpls.2019.00465

Wu, Y., Lee, S.-K., Yoo, Y., Wei, J., Kwon, S.-Y., Lee, S.-W., Jeon, J.-S., & An, G. (2018). Rice Transcription Factor OsDOF11 Modulates Sugar Transport by Promoting Expression of Sucrose Transporter and SWEET Genes. *Molecular Plant*, *11*(6), 833–845. https://doi.org/10.1016/j.molp.2018.04.002

Xiao, Q., Wang, Y., Li, H., Zhang, C., Wei, B., Wang, Y., Huang, H., Li, Y., Yu, G., Liu, H., Zhang, J., Liu, Y., Hu, Y., & Huang, Y. (2021). Transcription factor ZmNAC126 plays an important role in transcriptional regulation of maize starch synthesis-related genes. *The Crop Journal*, *9*(1), 192–203. https://doi.org/10.1016/j.cj.2020.04.014

Yang, S., Fu, Y., Zhang, Y., Peng Yuan, D., Li, S., Kumar, V., Mei, Q., & Hu Xuan, Y. (2022). Rhizoctonia solani transcriptional activator interacts with rice WRKY53 and grassy tiller 1 to activate SWEET transporters for nutrition. *Journal of Advanced Research*. https://doi.org/10.1016/j.jare.2022.10.001

Zhang, Y., Zhang, B., Yang, T., Zhang, J., Liu, B., Zhan, X., & Liang, Y. (2020). The GAMYB-like gene SlMYB33 mediates flowering and pollen development in tomato. *Horticulture Research*, *7*(1), 133. https://doi.org/10.1038/s41438-020-00366-1

Zhang, Z., Dong, J., Ji, C., Wu, Y., & Messing, J. (2019). NAC-type transcription factors regulate accumulation of starch and protein in maize seeds. *Proceedings of the National Academy of Sciences*, *116*(23), 11223–11228. https://doi.org/10.1073/pnas.1904995116
